# MicroRNA-494, Upregulated by Tumor Necrosis Factor-α, Desensitizes Insulin Effect in C_2_C_12_ Muscle Cells

**DOI:** 10.1371/journal.pone.0083471

**Published:** 2013-12-11

**Authors:** Hyunjoo Lee, Yuna Jee, Kyungki Hong, Gwi Seo Hwang, Kwang-Hoon Chun

**Affiliations:** 1 Gachon Institute of Pharmaceutical Sciences, College of Pharmacy, Gachon University, Incheon, Republic of Korea; 2 Lab of Cell Differentiation Research, College of Oriental Medicine, Gachon University, Seongnam, Republic of Korea; Tohoku University, Japan

## Abstract

Chronic inflammation is fundamental for the induction of insulin resistance in the muscle tissue of vertebrates. Although several miRNAs are thought to be involved in the development of insulin resistance, the role of miRNAs in the association between inflammation and insulin resistance in muscle tissue is poorly understood. Herein, we investigated the aberrant expression of miRNAs by conducting miRNA microarray analysis of TNF-α-treated mouse C_2_C_12_ myotubes. We identified two miRNAs that were upregulated and six that were downregulated by a >1.5-fold change compared to normal cells. Among the findings, qRT-PCR analysis confirmed that miR-494 is consistently upregulated by TNF-α-induced inflammation. Overexpression of miR-494 in CHO_IR/IRS1_ and C_2_C_12_ myoblasts suppressed insulin action by down-regulating phosphorylations of GSK-3α/β, AS160 and p70S6K, downstream of Akt. Moreover, overexpression of miR-494 did not regulate TNF-α-mediated inflammation . Among genes bearing the seed site for miR-494, RT-PCR analysis showed that the expression of *Stxbp5*, an inhibitor of glucose transport, was downregulated following miR-494 inhibition. In contrast, the expression of PTEN decreased in the cells analyzed, thus showing that both positive and negative regulators of insulin action may be simultaneously controlled by miR-494. To investigate the overall effect of miR-494 on insulin signaling, we performed a PCR array analysis containing 84 genes related to the insulin signaling pathway, and we observed that 25% of genes were downregulated (P<0.05) and 11% were upregulated (P<0.05). These results confirm that miR-494 might contribute to insulin sensitivity by positive and negative regulation of the expression of diverse genes. Of note, PCR array data showed downregulation of *Slc2A4*, a coding gene for Glut4. Altogether, the present study concludes that the upregulation of miR-494 expression by TNF-α-mediated inflammation exacerbates insulin resistance. Therefore, we suggest that miR-494 could prove an important target for the diagnosis and therapy of inflammation-mediated insulin resistance in muscle.

## Introduction

Insulin resistance, the condition in which normal insulin response is not produced by normal amounts of insulin, is a fundamental component of the pathogenesis of type 2 diabetes mellitus (T2D). Insulin resistance is associated directly with abdominal obesity, dyslipidemia, high blood sugar, hypertension, and coronary heart disease [1-3][1-3]. The causes of insulin resistance have not yet been fully deciphered, but inflammation and lipid overload have been considered as pivotal contributors [4]. In the lipid-overloaded hypothesis, accumulated fat inside the muscle cells and liver cells causes the buildup of diacylglycerols (DAGs) within cells, which results in a shut-down of insulin signaling [5]. The inflammation hypothesis, suggests that nutrient-overloaded fat cells release chemotactic adipokines followed macrophage infiltration, resulting in low-intensity chronic inflammation. Subsequently, the inflamed adipose tissue secretes inflammatory molecules into the blood stream, such as tumor necrosis factor-α (TNF-α) [6], interleukin-6 (IL-6) [7,8] and monocyte chemoattractant protein-1 (MCP-1) [9,10] that are thought to promote insulin resistance in the adipose, muscle, liver and pancreatic beta cells [11-16].

TNF-α was first described as an endotoxin-induced serum factor that induces necrosis of tumors [17]. TNF-α has deteriorative property on insulin resistance *in vivo*. The deteriorative ability of TNF-α on insulin resistance *in vivo* is evident from the fact that TNF-α is highly expressed in adipose tissue, muscle and serum of obese subjects [15,18-20] and obese animals [6,21,22]. Moreover, the treatment of TNF-α induces hepatic insulin resistance in obese Zucker rats [23]. In contrast, obese mice lacking either TNF-α or its receptors are protected from developing insulin resistance [24-26]. The heterozygous deletion of inhibitory-κB kinase β (IKKβ^+/-^) ameliorated diet-induced insulin resistance during high-fat feeding and in obese *Lep* ^*ob/ob*^ mice [27]. Several molecules have been suggested as targets of TNF-α-mediated insulin resistance. TNF-α regulates negatively insulin action by phosphorylation of serine residues on insulin receptor substrate-1 (IRS-1) via Ser307 by activated IKK [23,28], c-Jun N-terminal kinase (JNK) [29,30], mitogen-activated protein kinase/extracellular signal-regulated kinase kinase 1/2 (MEK1/2) [31], and mammalian target of rapamycin (mTOR) [32].[28,29] In 3T3-L1 adipocytes, TNF-α represses the transcription of glucose transporter type 4 (Glut4) [6,33]. Chronic treatment with TNF-α stimulates suppressor of cytokine signaling 3 (SOCS3) expression [34]. SOCS3 induces insulin resistance by directly binding to IRS-1 and promoting the ubiquitination and subsequent degradation of IRS-1 [35,36]. In addition, TNF-α can induce lipolysis in adipocytes [37], downregulate the activity of PPARγ and block differentiation of preadipocytes into adipocytes [38,39]. Troglitazone rescues the capacity for lipogenesis reduced by TNF-α in adipocytes via NF-κB inhibition [40]. However, more comprehensive studies are needed to fully understand the molecular mechanism whereby TNF-α induces insulin resistance. MicroRNAs (miRNAs) consist generally of 20-22 nucleotides that regulate gene expression [41]. MiRNA triggers downregulation of protein synthesis by deadenylating [42] and inhibiting translation [43,44] of target messenger RNAs (reviewed in 45,46). They are emerging as potential regulators of many pathological processes, including insulin resistance. Of particular relevance, upregulated miR-103 and miR-107 in obese mice alter hepatic insulin sensitivity [47]. Expression of miR-143 and miR-802 is upregulated in the liver of obese mice and impairs glucose metabolism [48]. Although diverse miRNAs have been reported as key regulators of insulin resistance, the mechanism by which miRNA associate inflammation signaling to the development of insulin resistance is not well studied. Therefore, we examined miRNAs dysregulated by chronic treatment of TNF-α in mouse C_2_C_12_ myotubes using miRNA microarray analysis. We found that miR-494 was upregulated by the TNF-α-induced inflammation and deteriorated insulin action in muscle cells.

## Materials and Methods

### Materials

Pre-miR^TM^-494 precursor was purchased from Ambion (Austin, TX, USA). Antibodies for IRS1, Akt, p-Akt (S473 and T308), p-AS160 (T642), p-ERK1/2 (T202/Y204), p-p38 (T180/Y182), IκB-α, p-NF-κB (S536), p-GSK-3α/β (S21/9), p-p70S6 kinase (T389), myogenin and myoD were purchased from Cell Signaling Technology, Inc. (Danvers, MA, USA). Antibodies for actin, α-tubulin, GAPDH, NF-κBp65, PTEN, ROCK1 (H85) were purchased from Santa Cruz Biotechnology, Inc. (Santa Cruz, CA, USA). Antibody for p-IRS1 (Y612) was from Millipore (Billerica, MA, USA).

### Cell culture and differentiation

Mouse C_2_C_12_ myoblasts, rat L6 myoblasts and CHO_IR/IRS1_ cells expressing the human insulin receptor and IRS1 (CHO_IR/IRS1_) were cultured in growth medium consisting of Dulbecco’s modified Eagle’s medium (DMEM) and Ham’s F-12 medium (Invitrogen, CA, USA) supplemented with 10% fetal bovine serum (Invitrogen) and 1% penicillin/streptomycin (Cellgro, VA, USA) in a humidified incubator at 37 °C and 5% CO_2_. Human HeLa cells were cultured in DMEM medium under the same conditions as above. Myotubes differentiation of C_2_C_12_ cells was induced after the cells reached confluence by replacing the growth medium with the differentiation medium (DMEM/ 2% horse serum (HS; Invitrogen)) which was changed every two days. 

### RNA isolation and gene expression profiling using microarray and PCR array

We performed global miRNA gene expression analyses using the Affymetrix GeneChip® miRNA Array. The array contains 46,228 probes comprising 7,815 probe sets, including controls. The content is derived from the Sanger miRbase miRNA database v11 (http://microrna.sanger.ac.uk). On Day five of differentiation, C_2_C_12_ myotubes were treated with 2 ng/ml TNF-α (Sigma-Aldrich) for four days replenishing the medium every two days. Total RNA was isolated using TRIzol, as described by the manufacturer (Ambion, USA) and 1ug of total RNA was used as the input for the labeling reaction and hybridized to analyze the Affymetrix GeneChip® miRNA Array. Images were scanned using a Genechip Array scanner 3000 7G (Affymetrix). The raw intensity data were further analyzed using R software.

For PCR array analysis, the 384-well Human Insulin Signaling Pathway PCR Array (SABiosciences) was used. This panel includes a probe for 84 insulin signaling-related genes and five housekeeping genes for PCR control. Total RNA was extracted from HeLa cells transfected with miR-494-mimic oligonucleotides using TRIzol and used for cDNA synthesis. Two independent cDNA stocks were prepared from each sample RT^2^ First Strand Kit (Qiagen), and each cDNA stock was independently analyzed in duplicate. A master mix was prepared using Roche LightCycler 480 SYBR Green I Master, 5 μl of which was added to each well of the panel plate. The Roche LightCycler 480 instrument was used for running real-time PCR according to the manufacturer’s instructions and for data analyses. PCR array data were calculated by the comparative cycle threshold method, normalized against multiple housekeeping genes and expressed as the mean fold change in microRNA-transfected samples relative to the control samples. Data analysis was conducted using a web-based data analysis tool from the PCR array manufacturer (http://sabiosciences.com/pcr/arrayanalysis.php).

### Reverse transcription PCR and quantitative real-time PCR

Total RNA from cells was extracted using the TRIzol reagent. For reverse transcription PCR (RT-PCR), cDNA was acquired from 500 ng of total RNA using SuperScript First-Strand Synthesis System for RT-PCR (Invitrogen, cat# 11904-018). Sequences of primers (mouse) used in PCR were as follows: *Pten*, forward 5’-aggccaaccgatacttctctc-3’, reverse 5’-catctggagtcacagaagttgaa-3’; *Rock1*, forward 5’-tgctaaccaaagatattgaaatgct-3’, reverse 5’-tttatttcttcctccttcttcaattt-3’; *mtTFA*, forward 5’-agggagctaccagaagcaga-3’, reverse 5’-cccatcagctgacttggagt-3’; *Igf1r*, forward 5’-ctctgaggccagaagtggag-3’, reverse 5’-ggtaggccatgccatctg-3’; *Irs1*, forward 5’-ctatgccagcatcagcttcc-3’, reverse 5’-ttgctgaggtcatttaggtcttc-3’; *Foxj3*, forward 5’-tttgaacagtgttggaagtgtacata-3’, reverse 5’-ttttctcgttctggaaggttg-3’; *Rab35*, forward 5’-agattcggactgtggagatca-3’, reverse 5’-catgggtcccccgataata-3’; *Stxbp5*, forward 5’-cggtgcacaatctctcgat-3’, reverse 5’-caccttcgatcccaccag-3’; *GAPDH*, forward 5’-accaccatggagaaggc-3’, reverse 5’-ggcatggactgtggtcatga-3’. Sequences of primers (human) used in PCR were as follows: *Gab1*, forward 5’-acctcaagccagacagaaaagt-3’, reverse 5’-tcgagcaaaactcctagtgatg -3’; *Grb2*, forward 5’-caagaactacatagaaatgaaaccaca-3’, reverse 5’-gataagaaaggccccatcgt-3’; *Insr*, forward 5’-aatgaacaggtgttgaaatttgtc-3’, reverse 5’-gggttgaattgccagcac-3’; *Jun*, forward 5’-gtgtttcgggagtgtccag-3’, reverse 5’-gtgggtaccgctgctttc-3’; *Ldlr*, forward 5’- gacctgttcccgacacctc-3’, reverse 5’-tcatttcctctgccagcaa-3’; *Ptpn1*, forward 5’-cggtcacttttgggagatg-3’, reverse 5’-gccagtattgtgcgcattt-3’; *Slc2A1* forward 5’-gtcaacacggccttcactg-3’, reverse 5’-ggtcatgagtatggcacaacc-3’; *Slc2A4*, forward 5’-ggcatgggtttccagtatgt-3’, reverse 5’-gcctcgagtttcaggtactctt-3’; *Gapdh* forward 5’-tttggtcgtattgggcgcctg-3’, reverse 5’-ccatgacgaacatgggggcat-3’. The PCR products were subjected to 1% agarose gel electrophoresis and the band intensity was measured and quantitated using the Image Lab 3.0 software (Bio-Rad).

Reverse transcription for qRT-PCR analysis was carried out from total RNA samples using the TaqMan®MicroRNA Reverse Transcription Kit (Invitrogen), and expression of the mature miRNA was measured with TaqMan®MicroRNA Assays (Invitrogen) using the Mx3005P qPCR System (Agilent Technologies, USA). The experiments were repeated at least three times, and the samples were analyzed in triplicate. The snoRNA202 and U6 snRNA were used as internal controls to normalize miRNA expression and the relative expression levels of miRNAs were compared by using the 2^-ΔΔCt^ method. 

### Transfection of miR-494 inhibitor and mimic

MiR-494 mimic (Pre-miR^TM^ miRNA Precursor, Ambion, cat# AM17100) and miR-494 antisense oligonucleotide (ASO) (Anti-miR^TM^ miRNA Inhibitor, cat# AM17000) were introduced into cells by transient transfection with a Lipofectamine RNAiMAX reagent and Lipofectamine 2000 (Invitrogen) according to the manufacturer’s instructions. Cells were grown to 50% confluence and 8 nM (final concentration) of both, the mimic and ASO, were used to transfect the cells. After two days, cells were treated with TNF-α or insulin at the indicated dose and duration as described in results. 

### PAGE and immunoblot analysis

Cells were suspended in lysis buffer (20 mM Tris pH 7.5, 5 mM EDTA, 10 mM Na_4_P_2_O_7_, 100 mM NaF, 2 mM Na_3_VO_4_, 1% NP-40, 1mM PMSF, 10 μg/ml aprotinin and 10 μg/ml leupeptin) and 20 μg of each fraction was separated by SDS-PAGE, followed by immunoblotting analysis. The membranes were incubated with antibodies and the bands were visualized with ChemiDoc imaging system (Bio-Rad) and quantified by Image Lab software (Bio-Rad).

### Statistical analysis

Data are expressed as means ± SEM. Comparison of the means was performed using unpaired Student’s t-test, and null hypotheses of no difference were rejected if p-values were less than 0.05. 

## Results

### TNF-α induced chronic inflammation in C_2_C_12_ myotubes

**Figure 1:**
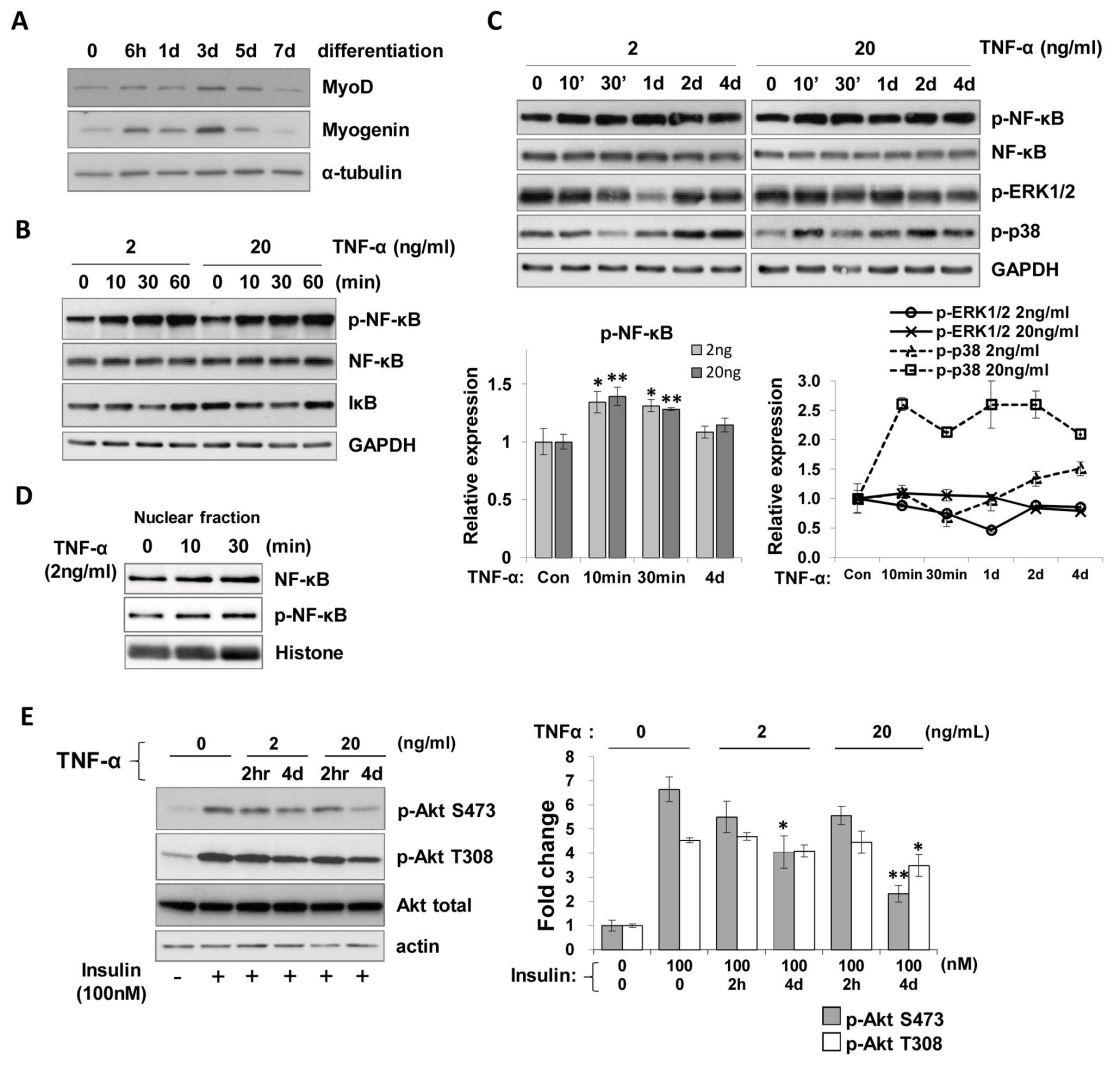
Induction of inflammation and insulin resistance by TNF-α treatment in C_2_C_12_ myotubes. (A) Differentiation of C_2_C_12_ myoblasts into myotubes was induced by replacing the growth medium with the differentiation medium (DMEM/ 2% horse serum). The levels of MyoD and Myogenin were measured by immunoblotting. α-tubulin was used as a loading control. (B-C) On Day 5 after differentiation, C_2_C_12_ cells were incubated with 2 ng/ml or 20 ng/ml TNF-α after starvation in serum-free DMEM as indicated. Cell lysates were subjected to immunoblot analysis. Bars and a line graph show densitometric quantitation of p-NF-κB (lower left), p-p38MAPK and p-ERK-1/2 (lower right) respectively. The values are expressed as the means ± SEM. Level of p-NF-κB after TNF-α stimulation was compared to that of untreated control. *,P<0.05; **,P<0.01. (D) Immunoblot assay for the nuclear translocation of NF-κB by TNF-α stimulation. Cells were treated with 2 ng/ml TNF-α and then the nuclear fraction was harvested and subjected to immunoblotting with antibodies to NF-κB and p-NF-κB. Histone was used as a loading control. (E) Induction of insulin resistance by TNF-α. Cells were treated with 100 nM insulin after pre-incubation with 2 or 20 ng/ml TNF-α in C_2_C_12_ myotubes for 2 h or 4 days. Insulin-induced p-Akt levels were measured by immunoblot analysis. Bars show densitometric quantitation of Akt phosphorylation. The values are expressed as the means ± SEM. *,P<0.05; **,P<0.01 versus TNF-α-untreated, insulin-stimulated control.

To examine the effects of inflammation on insulin resistance of muscle cells, we differentiated C_2_C_12_ cells into myotubes. The differentiation was qualified with morphology change and immunoblot assay by measuring MyoD and myogenin levels, differentiation markers, during myogenesis (Figure 1A). Immunoblot analysis showed that treatment with 2 ng/ml TNF-α in differentiated cells (day 5) resulted in increased phosphorylation of NF-κB (about 1.5 fold) at 60 min and decrease in IκB-α protein levels, demonstrating induction of inflammation (Figure 1B). No further increase in the levels of p-NF-κB was observed on treatment with a higher dose of TNF-α (20 ng/ml). Further, protein levels of NF-κB were not altered throughout the measurement time points whereas IκB-α protein levels were reduced by TNF-α treatment for 10 or 30 min and then restored to the control levels at 60 min, at both doses (Figure 1B). The activation of NF-κB was further confirmed with the observation of the nuclear forms of NF-κB and p-NF-κB after 2 ng/ml TNF-α treatment (Figure 1D). To investigate chronic effects of TNF-α on inflammatory signals, we treated cells with TNF-α for up to four days. We observed that NF-κB phosphorylation increased at an early stage (10 and 30 min), and gradually decreased to near basal state following prolonged incubation with TNF-α as shown in Figure 1C (upper and lower left). Previously, p38MAPK, extracellular signal-regulated kinase-1/2 (ERK-1/2) and c-Jun NH2-terminal kinase (JNK) mitogen-activated protein kinases (MAPKs) were shown to be required for the TNF-α-mediated disturbance of insulin signaling of muscle cells and endothelial cells [23,49,50]. When C_2_C_12_ myotubes were treated with a high dose of TNF-α (20 ng/ml), phosphorylation of p38MAPK increased and was sustained throughout the tests but, with low a dose of TNF-α, p-p38MAPK increased only with prolonged incubation as shown in Figure 1C (upper and lower right). In contrast, the level of p-ERK-1/2 decreased on incubation with TNF-α at 2 or 20 ng/ml and phosphorylation of both p38 and ERK-1/2 underwent fluctuations during TNF-α treatment in myotubes throughout the tests. These findings reveal that TNF-α has cellular effects at both 2 and 20 ng/ml that may involve signaling through the p38MAPK pathway while ERK1/2 may not be a major player.

### Insulin resistance deteriorated following chronic incubation of C_2_C_12_ myotubes with TNF-α

As TNF-α is a major mediator in the inflammatory process that is considered an inducer of insulin resistance (reviewed in 51,52), we investigated whether insulin signaling may be affected by TNF-α in C_2_C_12_ myotubes. As shown in Figure 1E, phosphorylation of Akt at the Ser473 residue increased significantly in insulin-stimulated C_2_C_12_ myotubes compared to controls, but was inhibited by approximately 20% following pre-incubation of 2 ng/ml TNF-α for 2 h. With prolonged incubation of 2 ng/ml TNF-α for 4 days, a further decrease in Akt phosphorylation at the Ser473 residue was observed (by 45%) compared to control. With a higher dose of TNF-α (20 ng/ml), additional decreases in p-Akt levels were observed at both Ser473 and Thr308 residues. These observations indicate that insulin resistance can be induced by acute and chronic states of inflammation, and that regulators other than NF-κB might additionally contribute to the deterioration of insulin resistance during chronic inflammation.

### Mir-494 expression increased during TNF-α-induced inflammation in C_2_C_12_ myotubes

**Figure 2:**
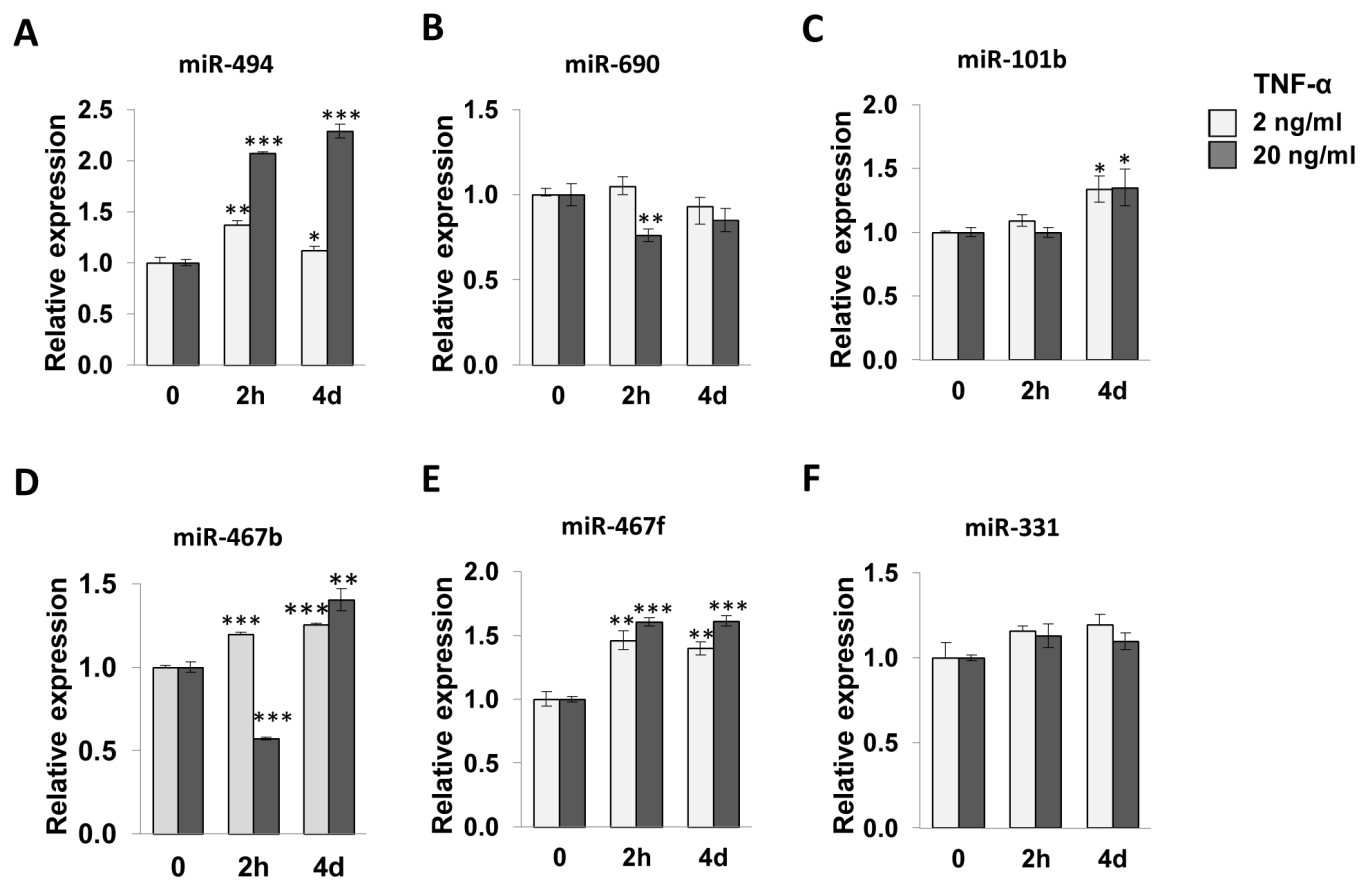
qRT-PCR analysis of miRNAs dysregulated in TNF-α treated C_2_C_12_ myotubes. Cells were treated with 2 or 20 ng/ml of TNF-α for 2 h or 4 days and the expression of miR-494, miR-690, miR-101b, miR-467b, miR-467f and miR-331 was measured by qRT-PCR. U6 RNA was used as a normalization control. The values are expressed as the means ± SEM. *,P<0.05; **,P<0.01; ***,P<0.001 versus vehicle-treated control.

To further investigate the mechanism of inflammation-mediated insulin resistance, we searched for microRNAs (miRNAs) that are deregulated in the inflammatory state evoked by TNF-α. For this purpose, we performed miRNA microarray analysis (miRbase v9.0) on C_2_C_12_ myotubes treated with 2 ng/ml TNF-α for 4 days to mimic low-grade and chronic inflammatory condition. We identified two upregulated miRNAs, namely miR-494 and miR-695 and six downregulated miRNAs, namely miR-331, miR-467b, miR-467f, miR-1195, miR-101b and miR-690 with statistical significance (p<0.05). The expression level of miR-494 increased 1.6-fold in TNF-α-treated cells. To verify the microarray data, qRT-PCR analysis was performed to quantify miRNA expression. We observed a 1.4-fold and 2-fold increase in miR-494 expression after 2 h incubation with 2 and 20 ng/ml TNF-α respectively, which decreased to 1.1-fold and 2.3-fold respectively, after 4 days of incubation in C_2_C_12_ myotubes (Figure 2A). The expression of miR-690 was decreased by TNF-α treatment as shown by microarray analysis (Figure 2B). In contrast to the microarray data, expression of miR-101 and miR-467f increased as observed by qRT-PCR, showing discrepancy between the two methods (Figures 2C and E), and miR-331 showed no significant change in expression (Figure 2F). Interestingly, miR-101b increased only at day four, indicating that this miRNA might be an effective regulator in the chronic state of inflammation. Based on these results, we further tested the effects of miR-494 because both methods demonstrated comparable results in the expression of the miRNA.

### MiR-494 antagonized insulin action in C_2_C_12_ myotubes and CHO_IR/IRS1_ cells

**Figure 3:**
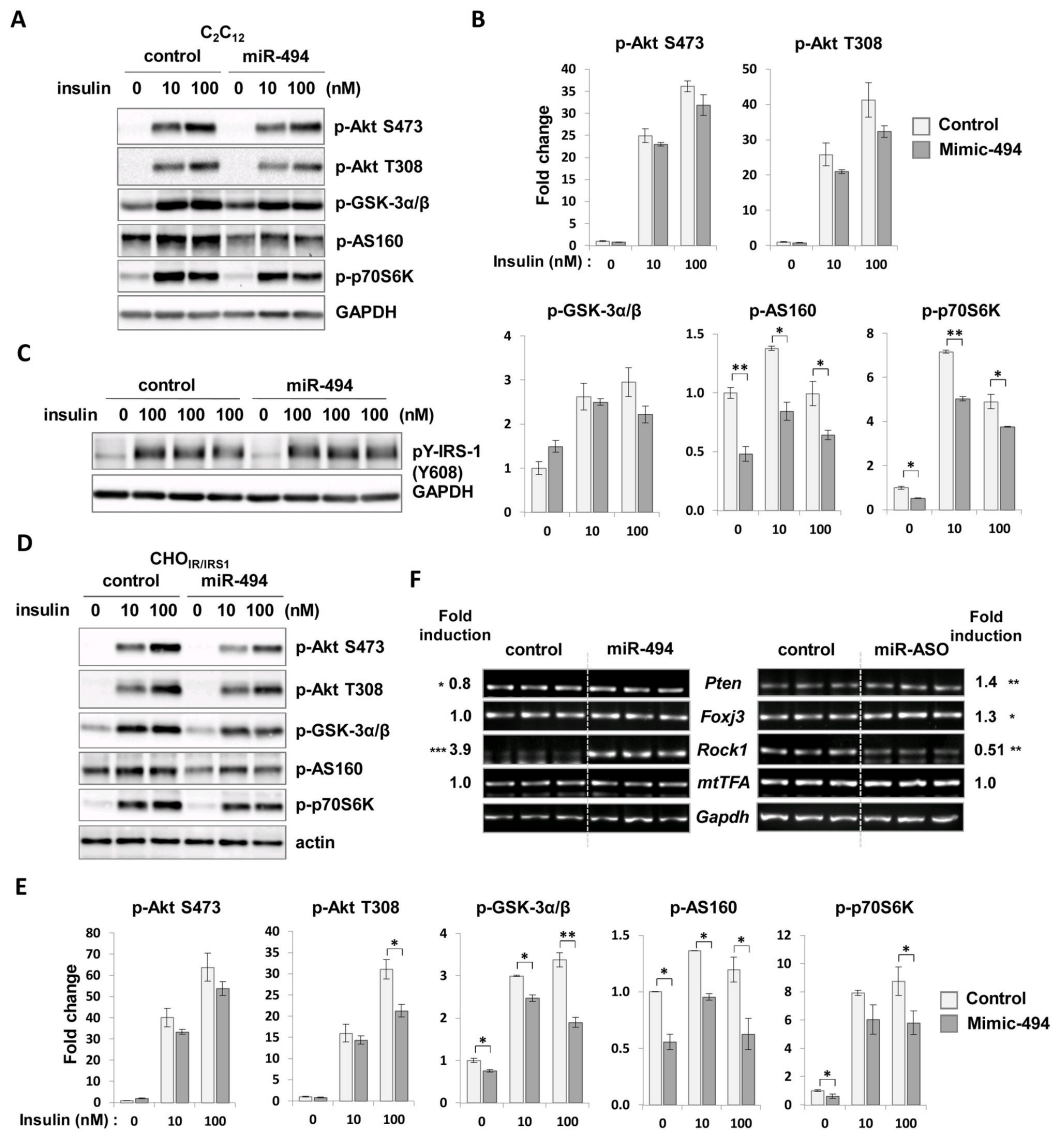
Effects of miR-494 on the insulin signaling pathway. The mimic form of miR-494 was overexpressed in cells. Cells were starved for 4 h before treatment with 0, 10, 100 nM of insulin for 20 min, and the levels of p-Akt (Ser473 and Thr308), p-AS160, p-p70S6K, and p-GSK-3α/β were measured in C_2_C_12_ myoblasts (A) and CHO_IR/IRS-1_ cells (D). Bars show densitometric quantitation of phosphorylations of Akt, GSK-3α/β, AS160 and p70S6K in C_2_C_12_ myoblasts (B) and CHO_IR/IRS-1_ cells (E). (C) The level of p-IRS1 (Tyr608) was measured in insulin-treated C_2_C_12_ myoblasts. GAPDH and actin were used as loading controls. Levels of phospho-proteins between miR-494 transfected cells and control were compared at each concentration of insulin. The values are expressed as the means ± SEM. *P<0.05; **,P<0.01. (F) RT-PCR analysis of genes regulated by miR-494. Total RNA from cells transfected with miR-494-mimic or ASO was prepared from C_2_C_12_ cells and subjected to reverse transcription and PCR. The three lanes represent the same sample with triplicate loading. Fold induction indicates the ratio of expression levels of mRNA in mimic- or ASO-transfected cells compared to control. The glyceraldehyde-3-phosphate dehydrogenase gene (Gapdh) was used as a loading control.

As TNF-α induced insulin resistance and miR-494 expression, we tested whether miR-494 can modulate insulin action. We treated 10 or 100 nM insulin in mimic of miR-494 transfected C_2_C_12_ myoblasts. Transfection efficiency was confirmed at about 80% using FAM-labeled control miRNA inhibitor without obvious toxicity (data not shown). In cells overexpressing miR-494, phosphorylation of Akt substrate of 160 kDa (AS160) and phosphoprotein 70 ribosomal protein S6 kinase (p70S6K), downstream targets of Akt were downregulated significantly (Figures 3A and B). Insulin-stimulated levels of phosphorylated Akt at Ser473 and Thr308 residues were decreased minimally and p-glycogen synthase kinase (GSK)-3α/β level did not change significantly in miR-494-overexpressing cells. Tyrosine phosphorylation of IRS-1 at residue 608 was unchanged (Figure 3C). Next, we further tested the effects of miR-494 overexpression on insulin signaling in CHO_IR/IRS1_ cells. As shown in Figures 3D and E, phosphorylation of AS160 and p70S6K were significantly downregulated by miR-494 overexpression as similar as in C_2_C_12_ myoblasts. Furthermore, significant reduction of p-GSK-3α/β level and p-Akt at T308 residue was observed in CHO_IR/IRS1_ cells transfected with miR-494. Altogether, these findings suggest that miR-494 has an insulin-desensitizing activity by downregulation of the activity of Akt. To confirm whether the miR-494 mimic displays cellular effects against insulin stimulation by accurately targeting its substrates, we measured the expression levels of previously known targets of miR-494 such as phosphatase and tensin homolog (PTEN) [53-55], Rho-associated protein kinase 1 (ROCK1) [56], mitochondrial transcription factor A (mtTFA) and forkhead box J3 (Foxj3) [57]. As shown in Figure 3F, reverse transcription PCR (RT-PCR) analysis showed that the *Pten* expression level was downregulated by ectopic expression of miR-494 in C_2_C_12_ cells and *Pten* and *Foxj3* expressions were increased following inhibition of miR-494. However, we did not observe any change in *mtTFA* expression in both miR-494 expressing and inhibited conditions. Moreover, contrary to a previous report [55], *Rock1* gene expression was upregulated in miR-494 expressing cells and downregulated in ASO-treated cells indicating that miR-494 might serve as an activator of gene expression for *Rock1*. Taken together, these data show that the mimic of miR-494 magnified the effect of endogenous miR-494 efficiently and with specificity.

### Mir-494 did not affect TNF-α-induced NF-κB activity in C_2_C_12_ cells

**Figure 4:**
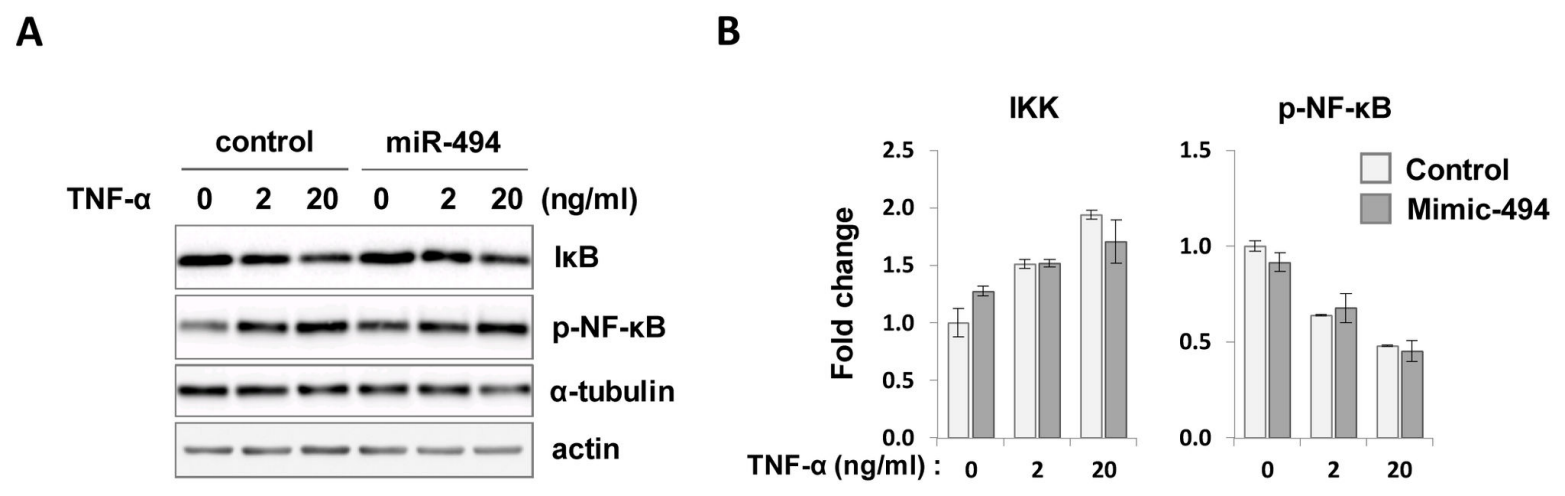
Effects of miR-494 on the inflammatory signaling. C_2_C_12_ myoblasts were transfected with miR-494 mimic oligonucleotides. (A) After 48 h of transfection, cells were starved and then treated with TNF-α at the indicated time and dose. Cell lysates were subjected to immunoblot analysis with antibodies to phospho-NF-κB and IκB. (B) Bars show densitometric quantitation of phosphorylations of NF-κB and IκB.

To evaluate whether miR-494 also affects TNF-α-induced inflammation, we transfected miR-494-mimic oligonucleotides into C_2_C_12_ myoblasts and treated with 2 or 20 ng/ml TNF-α for 30 min after overnight starvation. As shown in Figures 4A and B, TNF-α-induced phosphorylation levels of NF-κB and protein level of IκB were not changed by miR-494 overexpression compared to controls. These data indicate that miR-494 might not act as a positive or negative feedback controller in the TNF-α-induced inflammation. 

### Inhibition of miR-494 by antisense oligonucleotide (ASO) was not sufficient for induction of insulin action

**Figure 5:**
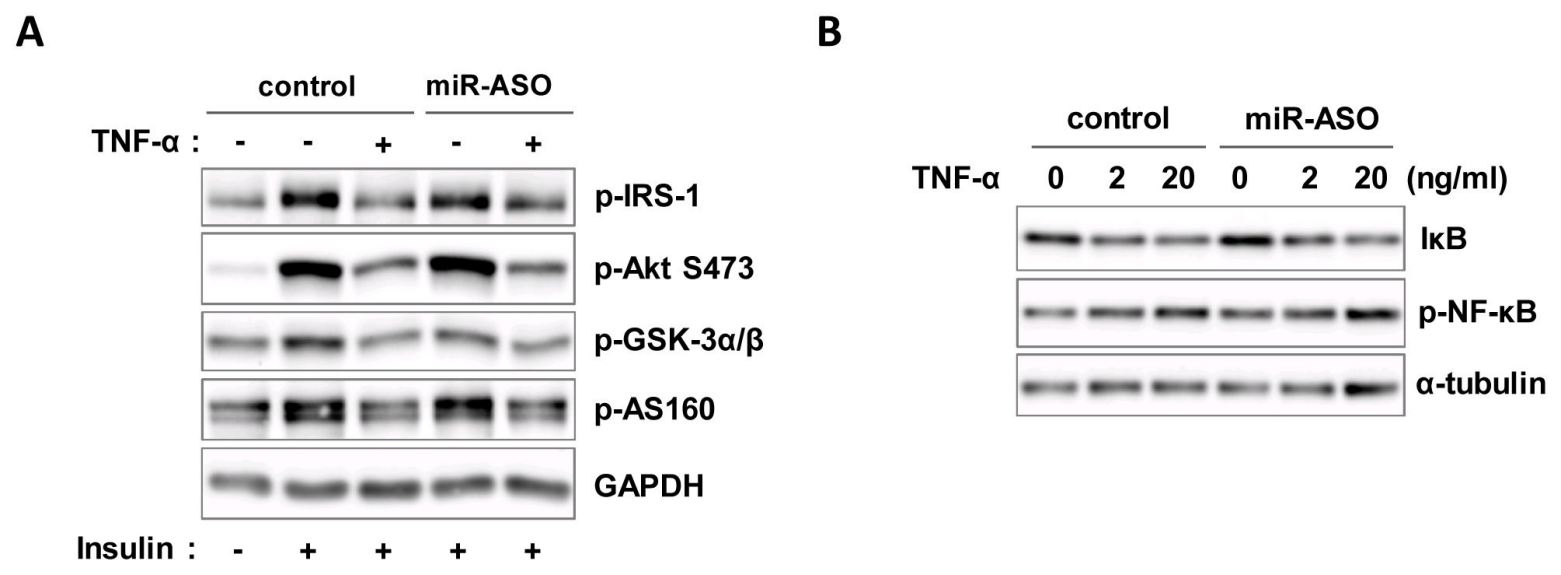
Effects of miR-494 inhibition on insulin and TNF-α signaling pathway. (A) C_2_C_12_ myoblasts transfected with miR-494 ASO were starved for 1 day and incubated with 20 ng/ml TNF-α for 1 day. Cells were treated with 100 nM insulin for 20 min. The levels of IRS-1, p-Akt (Ser473), and p-GSK-3α/β were measured. Bars show densitometric quantitation of phospho-proteins. (B) MiR-494-ASO transfected C_2_C_12_ myoblasts were incubated with TNF-α for 30 min. Cell lysates were prepared and subjected in immunoblot analysis using antibodies for p-NF-κB, NF-κB and IκB.

To verify the effect of miR-494 on insulin signaling, we inhibited miR-494 by transfecting ASO of miR-494 (miR-494-ASO) into C_2_C_12_ cells. Insulin action was significantly impaired by 20 ng/ml TNF-α pre-treatment, but inhibition of miR-494 could not restore the impairment as demonstrate by the similar levels of p-IRS-1, p-Akt and p-GSK-3α/β between miR-494-ASO transfected and control cells (Figure 5A). Further, inhibition of miR-494 did not affect TNF-α signaling (Figure 5B). Because the gene expression of *Pten, Foxj3* and *Rock1* were effectively regulated by miR-494-ASO in C_2_C_12_ cells (Figure 3F), it is possible that miR-494-ASO inhibits the action of miR-494 within cells, but inhibition of miR-494 is not sufficient for the activation of insulin signaling.

### *Stxbp5*, a predicted target gene, was partially downregulated by miR-494-ASO

**Figure 6:**
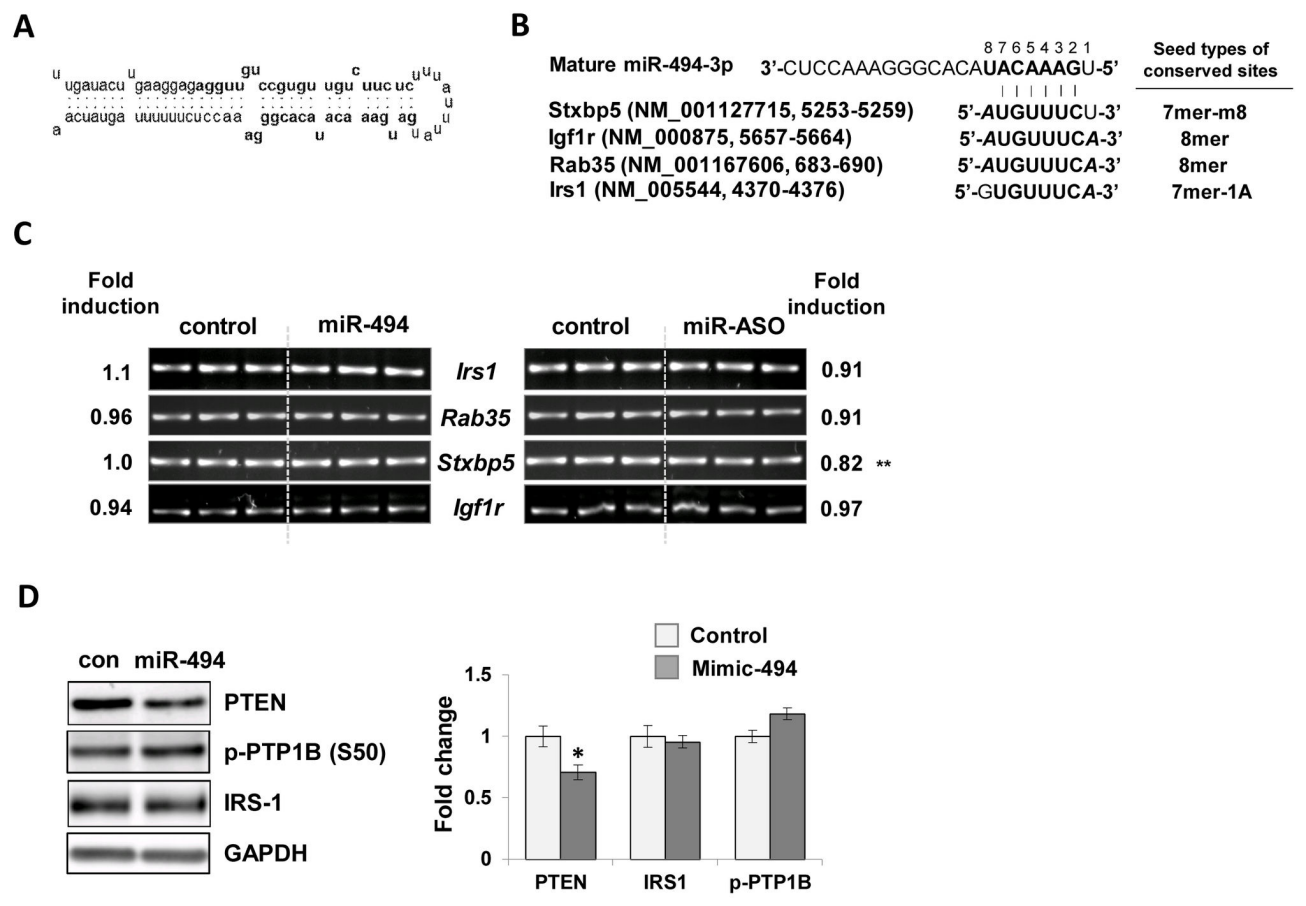
Regulation of putative target genes by miR-494. (A) The secondary structure of mouse premature(pre)-miR-494 (miRbase, Accession MI0003532). (B) Alignment of the seed matches in conserved sites of *Stxbp5, Igf1r, Rab35, Irs1* genes at 3’ untranslated region (3’ UTR) and seed types of each target gene. Entrez accession numbers and binding position at 3’ UTR are addressed for the representative transcript of each gene. Complementary sequences binding to miR-494 are shown in bold letters, with the m8 and A1 extensions highlighted in bold italic letters. Gene abbreviations: *Stxbp5*, syntaxin binding protein 5; *Igf1r*, insulin-like growth factor 1 receptor; Irs1, insulin receptor substrate 1 (C) RT-PCR analysis for predicted target genes in miR-494 mimic- or miR-494 ASO-transfected C_2_C_12_ cells. *Gapdh* gene was used as a loading control. (E) Immunoblot analysis of molecules related to the insulin signaling pathway. Cells were transfected with miR-494 mimic and subjected to immunoblot analysis. Antibodies for PTEN, p-PTP1B (S50) and IRS-1 were used. GAPDH was used as a loading control. The values were expressed as the means ± SEM and compared between miR-494 mimic transfected cells and control. *P<0.01.

To further identify the mechanism of insulin desensitizing effect of miR-494, we used TargetScan [58] and PITA (probability of interaction by target accessibility) [59] to identify targets of miR-494. The secondary structure of miR-494 is presented in Figure 6A. Of more than 350 conserved targets, we identified *Stxbp5* [60], *Igf1r* [61], *Rab35* [62], *Irs1* and *Pten* as probable regulators of the insulin signaling pathway, and identified the possible seed match between miR-494 and the 3’ untranslated region (UTR) of those genes (Figure 6B). Additionally, RT-PCR analysis was conducted to test if miR-494 can regulate the gene expression. Our data show that the mimic or ASO of miR-494 did not affect the expression of *Irs1, Rab35* and *Igf1r* (Figure 6C). *Stxbp5* gene alone was partially downregulated by miR-494-ASO in cells. Considering that *Stxbp5* is reported to inhibit Glut4 translocation, it is possible for miR-494 to regulate *Stxbp5* expression and in turn affect insulin stimulated Glut4 translocation. However, additional evidence is required to confirm whether *Stxbp5* is a direct target of miR-494 in cells because the miR-494-ASO-induced change was minimal (only a 20% decrease), and the gene expression was not altered by miR-494-mimic transfection.

### MiR-494 deteriorated insulin action despite inhibition of PTEN expression and PTP1B activity

PTP1B (protein-tyrosine phosphatase 1B) plays an important role in the regulation of insulin action by dephosphorylating the activated insulin receptor and downstream molecules such as insulin receptor and IRS-1. Because TNF-α-administration induces PTP1B expression of muscle in vivo [27] and PTP1B is inactivated by phosphorylation at Ser50 [63], we further tested whether miR-494 affects insulin action via activation of PTP1B. The phosphorylation of PTP1B at Ser50 was not decreased but partially increased in miR-494 transfected C_2_C_12_ cells compared to controls, indicating inhibition of insulin action may not be mediated by PTP1B activation (Figure 6D). From TargetScan prediction program [58] and PITA [59], we identified PTEN as a target involved in insulin signaling and PTEN was also found to be a target of miR-494 in previous reports [53-55]. As shown in Figure 6D, PTEN expression significantly decreased in miR-494 transfected C_2_C_12_ cells. Altogether, these findings suggest that miR-494 exacerbates insulin action through combined effects on the activity of multiple regulatory molecules in the insulin signaling pathway. 

### Mir-494 regulates expression of diverse genes related to the insulin signaling pathway

**Figure 7:**
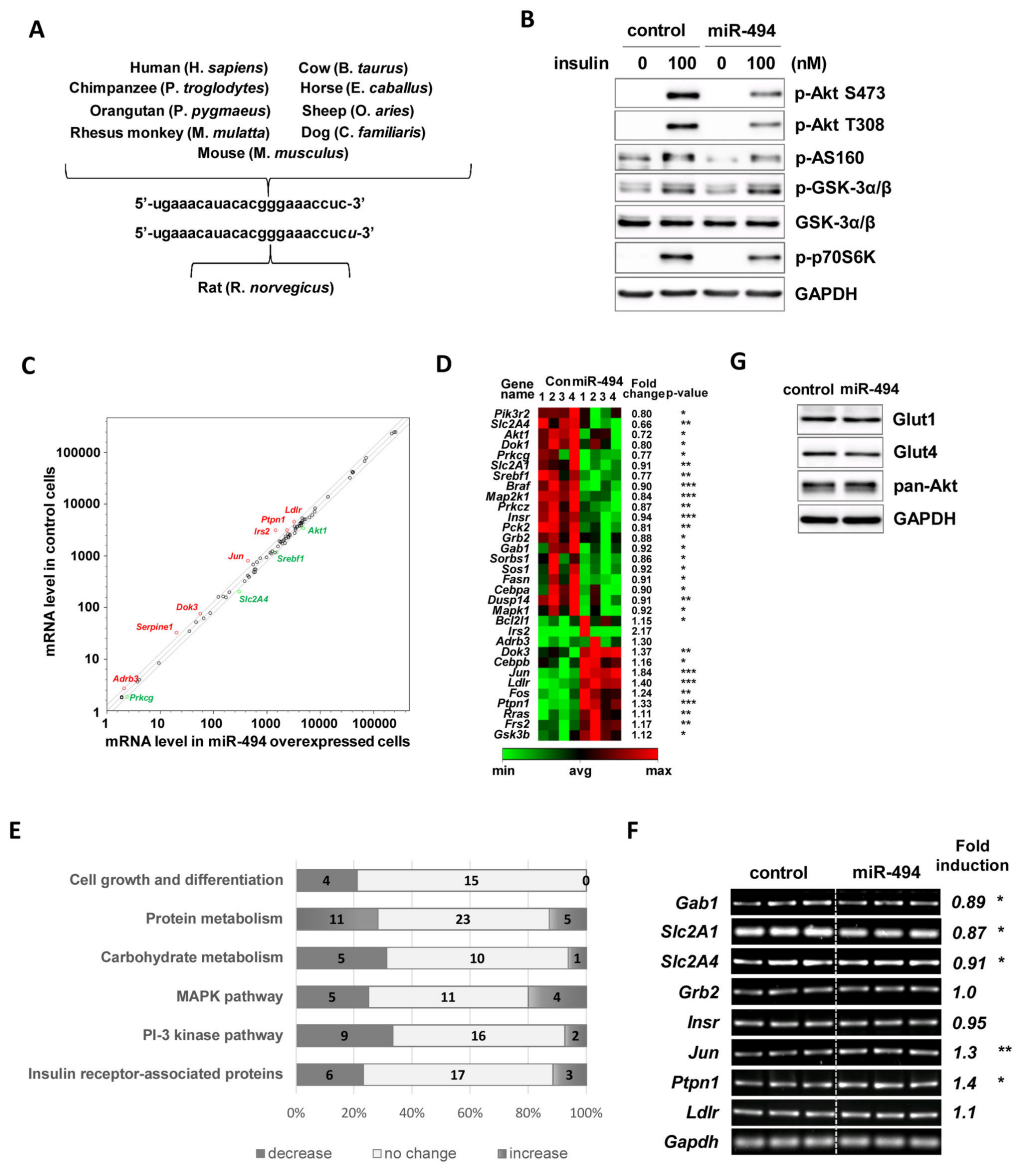
Regulation of genes related to the insulin signaling pathway by miR-494. (A) The sequences of mature form of miR-494 were compared between species. The sequences of miR-494 were obtained from miRbase (release 20; miRbase.org). (B) Human HeLa cells were overexpressed with the mimic form of miR-494. Cells were starved for 4 h before treatment with 100 nM of insulin for 20 min. The levels of p-Akt (Ser473 and Thr308), p-AS160, p-p70S6K, and p-GSK-3α/β were measured (C). Bars show densitometric quantitation of phosphorylations of Akt, GSK-3α/β, AS160 and p70S6K. GAPDH and actin were used as loading controls. (C-E) PCR array analysis of genes related to insulin signaling pathway by miR-494 in HeLa cells. Real-time PCR was carried out using Human Insulin Signaling Pathway PCR, after transfection of miR-494-mimic oligonucleotide. (C) Intensity scatter plot comparing the mRNA profiles of miR-494 transfected cells and control cells. The 11 mRNAs that were up- or downregulated more than 1.3-fold are highlighted and labeled. (D) Genes that changed more than 1.5-fold or with significance (P<0.05) were clustered. Gene names are denoted on the left. Fold changes and p-values are denoted on the right side of the panel. (E) The percentages of significantly (P<0.05) up- or downregulated genes are compared to the control in each metabolic category. The number of genes are described inside the bar in each group. (F) RT-PCR analysis was performed in miR-494 mimic transfected HeLa cells for 9 genes; *Slc2A4, Slc2A1, Insr, Grb2, Gab1, Ptpn1, Jun* and *Ldlr* with *Gapdh* as the loading control. The three lanes indicate the same sample with triplicate loading. Fold induction indicates the ratio of expression levels of mRNA in mimic transfected cells compared to control. Gene abbreviations: *Slc2A4*, solute carrier family 2 member 4; *Slc2A1*, solute carrier family 2 member 1; *Insr*, insulin receptor; Grb2, growth factor receptor-bound protein 2; *Gab1*, Grb2-associated binding protein 1; *Ptpn1*, protein tyrosine phosphatase, non-receptor type 1; *Jun*, jun proto-oncogene and *Ldlr*, low density lipoprotein receptor. (G) Immunoblot analysis of Glut1 and Glut4 was performed in miR-494 mimic transfected HeLa cells. GAPDH was used as a loading control.

The fact that the sequence of mature miR-494 is well conserved between most species (Figure 7A) suggests that the role of miR-494 in insulin action might be conserved well in humans too. To verify this possibility, we tested the effect of miR-494 on insulin sensitivity in human cells by transfecting mimic oligonucleotide into HeLa cells. By the ectopic expression of miR-494 in HeLa cells, the insulin-stimulated increases of p-Akt, p-p70S6K and p-AS160 were downregulated, indicating that the effect of miR-494 on insulin signaling pathway is well conserved in humans (Figure 7B). 

Because our findings indicate that the insulin desensitizing effect of miR-494 might be stem from the combined regulation of various targets related to insulin signaling, we further elucidated the mechanisms underlying miR-494-mediated insulin desensitization by conducting a PCR array experiment which consists of a panel of 84 genes related to the insulin signaling pathway. MiR-494 overexpression caused a significant change in the expression levels of 16 mRNAs (P<0.01). All genes were grouped by their function in the insulin signaling pathway and addressed with fold changes in p-values (Table S1). Of the differentially expressed genes, *Slc2A4, Akt1, Prkcg* and *Srebf1* were downregulated and *Jun, Ldlr, Dok3* and *Ptpn1* were upregulated more than 1.4 fold (Figure 7C). *Irs2* and *Adrb3* were significantly upregulated. The heat map of 32 genes with a greater than 1.3-fold or a significant (P<0.05) change was generated and labeled with fold changes and p-values (Figure 7D). The number of downregulated genes was twice that of upregulated genes (downregulated : upregulated : no change = 21 : 9 : 54). When 84 genes were grouped according to their role in insulin signaling, the percentage of downregulated genes was highest among cell growth-related genes and PI-3 kinase pathway-related genes as shown in Figure 7E. These data suggest that downregulation of the PI-3 kinase pathway might be one of the major causes of miR-494-mediated insulin desensitization. Among genes related to the PI-3 kinase pathway, *Slc2A4* and *Akt1* were downregulated by miR-494 with fold changes of 0.66 (P<0.5) and 0.72 (P<0.01) respectively. Because Akt1 participates in signaling of cell survival pathways and Akt2 is associated with insulin signaling, downregulation of Akt1 by miR-494 may not be a major contributor to the regulation of insulin sensitivity. On the other hand, the *Slc2A4* gene codes for Glut4 which functions as an insulin-regulated facilitative glucose transporter in adipose tissue and skeletal muscle. Further, *Insr* (0.94-fold, P<0.001)*, Grb2* (0.88-fold, P<0.05)*, Gab1* (Grb2-associated binding protein 1) (0.92-fold, P<0.05), and *Srebf1* (0.77-fold, P<0.01) were downregulated, suggesting that miR-494 might also inhibit insulin-stimulated cell growth via Ras-Raf-MAPK pathway and lipid biosynthesis. To confirm this, we measured the expression levels of *Slc2A1, Slc2A4, Insr, Grb2, Gab1, Jun, Ldlr* and *Ptpn1* in miR-494 mimic-transfected cells by RT-PCR analysis (Figure 7F). We found that *Gab1* (0.89-fold, P<0.05)*, Slc2A1* (0.87-fold, P<0.05) and *Slc2A4* (0.91-fold, P<0.05) are downregulated and *Jun* (1.3-fold, P<0.01) and *Ptpn1* (1.4-fold, P<0.05) are upregulated significantly in miR-494-mimic transfected cells while *Grb2* and *Ldlr* expression was not altered. Of note, the ectopic expression of miR-494 significantly downregulated Glut1 and Glut4 protein expression without change in total expression level of Akt isoforms (Figure 7G). These data indicate that miR-494 may play an important role in insulin resistance by downregulation of Glut4-mediated glucose transport in insulin-sensitive cells, in addition to the downregulation of PI-3K-Akt signaling pathway. 

Altogether, these findings demonstrate that increased miR-494 following inflammation exacerbates insulin sensitivity by inactivating the insulin signaling pathway and downregulating *Slc2A4* gene expression. Thus, we suggest that miR-494 is an attractive target for research in the prevention of inflammation-mediated insulin resistance.

## Discussion

The chronic, low-grade inflammation signal is hypothesized as a significant cause of insulin resistance in muscle [4,64]. Recently, miRNA-regulatory networks were discovered to have a key physiological role in the development of diseases. In the current study, we investigated novel small RNAs mediating insulin resistance raised by the inflammation stimulus, using a miRNA microarray. We invoked chronic, low-grade inflammation by exposing C_2_C_12_ myotubes to a relatively low level of TNF-α for 4 days, as the biophysical effects of TNF-α and its downstream signaling network were well established by the induction of insulin resistance in various animal models [24,25,29,65]. Our results demonstrated that miR-494 is strongly associated with the development of inflammation-mediated insulin resistance. Our data showed that miR-494 was upregulated by TNF-α and the overexpression of miR-494 into muscle cells conferred as insulin desensitizing effect. Therefore, based on these data, we suggest that inflammation triggers an increase in miR-494 expression resulting in exacerbation of insulin sensitivity. Additionally, it seems that miR-494 desensitizes the insulin signaling pathway by itself independent of the onset of inflammation.

Insulin resistance in insulin sensitive tissues is an early pathophysiologic feature in T2D, and interventions to improve insulin sensitivity are of paramount important for the cure of T2D [66]. Because muscle tissue accounts for over 85-90% of the impairment in total body glucose disposal in individuals with T2D [67,68], understanding of the mechanism of insulin resistance in muscle tissue are of importance for the prevention and cure of T2D. In terms of adipose tissue and liver, several studies suggested important roles of microRNAs in insulin resistance [69-72]. For example, mir-29 is upregulated in diabetic rats and is linked to insulin resistance in 3T3-L1 adipocytes [69]. The effects of miR-29 were suggested to be mediated through the downregulation of key mediators of the insulin signaling pathway such as caveolin 2 (CAV2) and insulin-induced gene 1 (INSIG1). Two other miRNAs, miR-133 and miR-320, also reduce insulin resistance by regulation of glucose transport in adipocytes [70,71]. Recently, miR-103 and miR-107 were reported to be upregulated in the livers of leptin-deficient (ob/ob) and diet-induced obese (DIO) mice and antisense therapy improved insulin sensitivity and glucose homeostasis in mouse models; thus microRNAs are attractive therapeutic targets in insulin resistance [47]. In contrast, microRNAs are rarely reported in the muscle cells or tissue. One study showed that miR-133 and miR-126 regulate GLUT4 transcription and translocation into the plasma membrane in rat muscle tissue [70,73]. In this regard, our report provides important information in that a novel miRNA was identified that can explain the mechanism of insulin resistance in the muscle system; additionally, a new link associating insulin resistance to inflammation was discovered.

Recent studies have reported that the mechanism of regulation of miR-494 expression and also that the miRNA plays a relevant role during tumorigenesis. Mir-494 was introduced as an oncogenic microRNA to promote tumorigenesis in hepatocellular carcinoma [74], breast cancer by activating myeloid-derived suppressor cells (MDSCs) [75], and non-small-cell lung cancer (NSCLC) cells by inducing Tumor necrosis factor-related apoptosis-inducing ligand (TRAIL) resistance [56]. In contrast, others showed that miR-494 acts as a tumor suppressor in lung cancer cells [76], human diploid fibroblasts [77], and gastrointestinal stromal tumor [78] by triggering apoptosis. These contradictory cellular effects of miR-494 in cell growth and death might, in part, result from intricate and divergent regulatory networks affected by miR-494 based on cell type. Interestingly, Yamamoto et al., showed that miR-494 was downregulated during C_2_C_12_ myocyte differentiation and skeletal muscle adaptation to physical exercise. They suggested *mtTFA* and *Foxj3* as putative target genes for miR-494 [57]. Along with this finding, Greco et al., reported increased muscle expression of miR-494 in patients with Duchenne muscular dystrophy (DMD) [79]. Here, we introduce miR-494 as a novel modulator of insulin sensitivity in an *in vitro* system.

Although miR-494 has been shown to directly regulate several target genes, such as *Pten* [55,75], *Cdk6* [80] and *Igf2bp1* [55], the role of miR-494 in terms of insulin sensitivity was yet unknown. For this reason, we examined the targets of miR-494 using prediction programs TargetScan and PITA and identified a few genes related to insulin signaling such as *Stxbp5*, *Igf1r*, *Rab35, Irs1, Rock1* and *Pten*. Our RT-PCR and immunoblot assays suggest that miR-494 positively and negatively regulates gene expression related to insulin action, and affects insulin signaling through combined effect of these factors. This idea is further supported by the result from a PCR array experiment using the insulin signaling pathway panel (Figure 7). Wang et al., also reported that miR-494 targets both proapoptotic and antiapoptotic proteins, leading to cardioprotective effects against ischemia/reperfusion (I/R)-induced injury in a mouse model [55]. *Stxbp5* encodes a syntaxin binding protein 5 (Tomosyn), which is a cytosolic Syntaxin1A-binding protein involved in insulin-stimulated Glut4 translocation. The binding of Tomosyn with Syntaxin1A interferes assembly of the SNARE complex formation, thus resulting in the inhibition of insulin-stimulated Glut4 translocation [60]. Rab35 also mediates insulin-stimulated Glut4 translocation positively in adipocytes [62]. Our study shows that *Stxbp5* expression is downregulated by miR-494-ASO. Considering that miR-494-ASO did not change Akt-related signaling (Figure 4), and *Stxbp5* expression was not affected by mimic of miR-494 (Figure 7C), we speculate that *Stxbp5* is not the primary contributor related to miR-494-induced insulin desensitization. To confirm *Stxbp5* as an authentic target of miR-494 in the insulin signaling pathway, further studies such as luciferase reporter assay are needed. A previous report suggested that ROCK1 and PTEN are downregulated in a mouse model when the cardiac-specific expression of miR-494 and Akt signaling pathway were induced [55]. Similarly, we observed that PTEN was downregulated following the ectopic expression of miR-494 in tested cells. However, our study shows that ROCK1 is upregulated and Akt signaling inactivated following insulin stimulation. Because ROCK1 was reported as a direct target using the luciferase reporter assay, it is surprising that ROCK1 was induced in our study. Various experimental models, such as murine ischemia/reperfusion-injured and human infarcted hearts [55], myeloid-derived suppressor cells (MDSCs) [75] and malignant transformed human bronchial epithelial cells, 16HBE [53], identified PTEN as a target of miR-494. Interestingly, our study shows that the insulin effect was negatively regulated in miR-494 overexpressing cells, although miR-494 reduces the expression of PTEN in C_2_C_12_ cells. Altogether, with the PCR array experiment, our data show that miR-494 may have various targets among the regulatory molecules of the insulin signaling pathway, and the effects are revealed by a complex interplay between signals.

Studies have suggested that TNF-α inactivates IRS1 by phosphorylating its Ser307 residue in skeletal muscle through activation of stress kinases such as JNK [29,30], ERK1/2 [31] or p38MAPK [23]. However, our study shows ERK1/2 might not be the main factor regulating the chronic state of insulin resistance at least in the tested cells (Figure 1C). We found that overexpression of miR-494 had no significant effect on the activity of IRS-1 as revealed by no change in the phosphorylation at the tyrosine residue (Figure 3C). This observation suggests that the mechanism of action of miR-494 might not be primarily mediated through MAPK pathway, leading to the downregulation of IRS-1. In addition, our immunoblot analysis shows that NF-κB and IκB may not be major contributors to the chronic state of inflammation of muscle cells nor the target of miR-494, as demonstrated in Figure 4. 

Our results from RT-PCR and PCR array analysis demonstrate that miR-494 positively and negatively affects a multitude of regulators. We speculate that the effect of miR-494 on insulin signaling is a result of the combined action of these various regulators. Nevertheless, our data suggest that the role of miR-494 is well conserved and *Slc2A4* gene would be a promising effector regulated by miR-494 associated with the deterioration of insulin action in adipose tissue or muscle.

## Supporting Information

Table S1 **Changes in gene expression after miR-494 treatment of HeLa cells.** RNA from HeLa cells transfected with miR-494 mimic oligonucleotide was used for insulin signaling pathway PCR array. Assay was performed in quadruplicate for each group.
(DOCX)
