# Supplementary material for: MicroRNA-494, Upregulated by Tumor Necrosis Factor-α, Desensitizes Insulin Effect in C2C12 Muscle Cells
Source: PLoS One. 2013 Dec 11;8(12):e83471. doi: 10.1371/journal.pone.0083471 (PMC3859653; doi:10.1371/journal.pone.0083471)
Supplement: Table S1 — Changes in gene expression after miR-494 treatment of HeLa cells. RNA from HeLa cells transfected with miR-494 mimic oligonucleotide was used for insulin signaling pathway PCR array. Assay was performed in quadruplicate for each group. (DOCX) [file pone.0083471.s001.docx]

Table S1. Changes in gene expression after miR-494 treatment of HeLa cells

| **Categories** | **gene symbol** | **fold change** | **95% CI** | **p-value** | |
| --- | --- | --- | --- | --- | --- |
| **IR-associated proteins** | DOK1 | 0.7994 | ( 0.66, 0.94 ) | 0.038178 | * |
|  | SORBS1 | 0.8612 | ( 0.76, 0.96 ) | 0.04568 | * |
|  | GRB2 | 0.8809 | ( 0.79, 0.97 ) | 0.040764 | * |
|  | GAB1 | 0.9167 | ( 0.86, 0.98 ) | 0.042123 | * |
|  | FRS3 | 0.9343 | ( 0.60, 1.27 ) | 0.711973 |  |
|  | INSR | 0.9408 | ( 0.92, 0.96 ) | 0.000896 | *** |
|  | INSR | 0.9408 | ( 0.92, 0.96 ) | 0.000896 | *** |
|  | INS | 0.9507 | ( 0.88, 1.02 ) | 0.231797 |  |
|  | IRS4 | 0.9507 | ( 0.88, 1.02 ) | 0.231797 |  |
|  | IGF2 | 0.9507 | ( 0.88, 1.02 ) | 0.231797 |  |
|  | IGFBP1 | 0.9507 | ( 0.88, 1.02 ) | 0.231797 |  |
|  | DOK2 | 0.9507 | ( 0.88, 1.02 ) | 0.231797 |  |
|  | PDPK1 | 0.9673 | ( 0.91, 1.03 ) | 0.344826 |  |
|  | INSL3 | 0.9774 | ( 0.45, 1.51 ) | 0.7962 |  |
|  | PTPRF | 0.9859 | ( 0.93, 1.05 ) | 0.648232 |  |
|  | IGF1R | 0.9927 | ( 0.94, 1.05 ) | 0.794456 |  |
|  | CBL | 0.9962 | ( 0.93, 1.06 ) | 0.89076 |  |
|  | PPP1CA | 0.9962 | ( 0.85, 1.14 ) | 0.948876 |  |
|  | NCK1 | 1.0084 | ( 0.93, 1.09 ) | 0.863446 |  |
|  | SHC1 | 1.0154 | ( 0.95, 1.08 ) | 0.645259 |  |
|  | CAP1 | 1.0457 | ( 0.96, 1.13 ) | 0.322959 |  |
|  | IRS1 | 1.0882 | ( 0.94, 1.23 ) | 0.266859 |  |
|  | FRS2 | 1.1724 | ( 1.09, 1.26 ) | 0.004405 | ** |
|  | PTPN1 | 1.3328 | ( 1.23, 1.44 ) | 0.000476 | *** |
|  | DOK3 | 1.3679 | ( 1.17, 1.56 ) | 0.002222 | ** |
|  | IRS2 | 2.1652 | ( 0.00001, 4.65 ) | 0.324572 |  |
| **PI-3 kinase pathway** | SLC2A4 | 0.6641 | ( 0.52, 0.80 ) | 0.007827 | * |
|  | AKT1 | 0.7167 | ( 0.56, 0.88 ) | 0.018722 | ** |
|  | SREBF1 | 0.7708 | ( 0.69, 0.85 ) | 0.00294 | ** |
|  | PRKCG | 0.7749 | ( 0.63, 0.92 ) | 0.037262 | * |
|  | PIK3R2 | 0.8008 | ( 0.67, 0.93 ) | 0.032871 | * |
|  | PCK2 | 0.812 | ( 0.75, 0.88 ) | 0.001783 | ** |
|  | PRKCZ | 0.8672 | ( 0.82, 0.92 ) | 0.002485 | ** |
|  | UCP1 | 0.8947 | ( 0.27, 1.52 ) | 0.527879 |  |
|  | DUSP14 | 0.9072 | ( 0.86, 0.95 ) | 0.008593 | ** |
|  | SLC2A1 | 0.9119 | ( 0.88, 0.95 ) | 0.00296 | ** |
|  | AKT3 | 0.9263 | ( 0.83, 1.02 ) | 0.207197 |  |
|  | EIF4EBP1 | 0.9343 | ( 0.84, 1.03 ) | 0.231111 |  |
|  | PRKCI | 0.9343 | ( 0.87, 1.00 ) | 0.087155 |  |
|  | MTOR | 0.9474 | ( 0.89, 1.01 ) | 0.1352 |  |
|  | G6PC | 0.9507 | ( 0.88, 1.02 ) | 0.231797 |  |
|  | IGFBP1 | 0.9507 | ( 0.88, 1.02 ) | 0.231797 |  |
|  | VEGFA | 0.9573 | ( 0.92, 0.99 ) | 0.061682 |  |
|  | EIF2B1 | 0.9589 | ( 0.90, 1.02 ) | 0.234024 |  |
|  | PDPK1 | 0.9673 | ( 0.91, 1.03 ) | 0.344826 |  |
|  | PIK3CA | 0.9997 | ( 0.92, 1.08 ) | 0.950905 |  |
|  | GSK3A | 1.0084 | ( 0.95, 1.07 ) | 0.79327 |  |
|  | PIK3R1 | 1.0101 | ( 0.91, 1.11 ) | 0.875045 |  |
|  | HK2 | 1.0101 | ( 0.92, 1.10 ) | 0.860437 |  |
|  | AKT2 | 1.0207 | ( 0.97, 1.08 ) | 0.477315 |  |
|  | GSK3B | 1.1227 | ( 1.04, 1.20 ) | 0.01837 | * |
|  | BCL2L1 | 1.1543 | ( 1.03, 1.27 ) | 0.038533 | * |
|  | SERPINE1 | 1.596 | ( 0.96, 2.23 ) | 0.063175 |  |
| **MAPK pathway** | MAP2K1 | 0.8348 | ( 0.79, 0.88 ) | 0.000422 | *** |
|  | GRB2 | 0.8809 | ( 0.79, 0.97 ) | 0.040764 | * |
|  | UCP1 | 0.8947 | ( 0.27, 1.52 ) | 0.527879 |  |
|  | BRAF | 0.9041 | ( 0.88, 0.93 ) | 0.000521 | *** |
|  | ANG | 0.9056 | ( 0.62, 1.19 ) | 0.615926 |  |
|  | SOS1 | 0.9151 | ( 0.86, 0.97 ) | 0.029391 | * |
|  | MAPK1 | 0.9231 | ( 0.88, 0.97 ) | 0.022478 | * |
|  | ERCC1 | 0.9295 | ( 0.80, 1.06 ) | 0.352277 |  |
|  | RAF1 | 0.949 | ( 0.90, 1.00 ) | 0.117423 |  |
|  | EIF2B1 | 0.9589 | ( 0.90, 1.02 ) | 0.234024 |  |
|  | RPS6KA1 | 0.9673 | ( 0.92, 1.02 ) | 0.24085 |  |
|  | HRAS | 0.969 | ( 0.81, 1.13 ) | 0.724884 |  |
|  | SHC1 | 1.0154 | ( 0.95, 1.08 ) | 0.645259 |  |
|  | ARAF | 1.0313 | ( 0.87, 1.19 ) | 0.728604 |  |
|  | RRAS2 | 1.064 | ( 1.01, 1.12 ) | 0.054366 |  |
|  | RRAS | 1.1053 | ( 1.05, 1.16 ) | 0.005664 | ** |
|  | NOS2 | 1.113 | ( 0.96, 1.27 ) | 0.177162 |  |
|  | BCL2L1 | 1.1543 | ( 1.03, 1.27 ) | 0.038533 | * |
|  | FOS | 1.2393 | ( 1.13, 1.35 ) | 0.003102 | ** |
|  | LDLR | 1.404 | ( 1.32, 1.49 ) | 0.000024 | *** |
| **Primary target genes for insulin signaling** | CEBPA | 0.8978 | ( 0.82, 0.97 ) | 0.047695 | * |
|  | PRL | 0.9507 | ( 0.88, 1.02 ) | 0.231797 |  |
|  | CEBPB | 1.1603 | ( 1.05, 1.27 ) | 0.022881 | * |
|  | FOS | 1.2393 | ( 1.13, 1.35 ) | 0.003102 | ** |
|  | LEP | 1.2874 | ( 1.04, 1.53 ) | 0.040359 | * |
|  | JUN | 1.8397 | ( 1.69, 1.99 ) | 0.000002 | *** |
| **Secondary effector target genes for insulin signaling** | NPY | 0.9825 | ( 0.91, 1.05 ) | 0.631675 |  |
|  | SLC2A4 | 0.6641 | ( 0.52, 0.80 ) | 0.007827 | ** |
|  | PCK2 | 0.812 | ( 0.75, 0.88 ) | 0.001783 | ** |
|  | SLC2A1 | 0.9119 | ( 0.88, 0.95 ) | 0.00296 | ** |
|  | PRL | 0.9507 | ( 0.88, 1.02 ) | 0.231797 |  |
| **Target genes for PPARγ** | PCK2 | 0.812 | ( 0.75, 0.88 ) | 0.001783 | ** |
|  | CEBPA | 0.8978 | ( 0.82, 0.97 ) | 0.047695 | * |
|  | ACOX1 | 0.9457 | ( 0.86, 1.03 ) | 0.245107 |  |
|  | RETN | 0.9507 | ( 0.88, 1.02 ) | 0.231797 |  |
|  | AEBP1 | 0.9842 | ( 0.84, 1.13 ) | 0.875177 |  |
|  | CAP1 | 1.0457 | ( 0.96, 1.13 ) | 0.322959 |  |
|  | PPARG | 1.0677 | ( 1.00, 1.13 ) | 0.077831 |  |
|  | CEBPB | 1.1603 | ( 1.05, 1.27 ) | 0.022881 | * |
|  | ADRB3 | 1.3009 | ( 0.86, 1.74 ) | 0.20485 |  |
| **Transcription factors and regulators** | SREBF1 | 0.7708 | ( 0.69, 0.85 ) | 0.00294 | ** |
|  | CEBPA | 0.8978 | ( 0.82, 0.97 ) | 0.047695 | * |
|  | AEBP1 | 0.9842 | ( 0.84, 1.13 ) | 0.875177 |  |
|  | CBL | 0.9962 | ( 0.93, 1.06 ) | 0.89076 |  |
|  | PPARG | 1.0677 | ( 1.00, 1.13 ) | 0.077831 |  |
|  | CEBPB | 1.1603 | ( 1.05, 1.27 ) | 0.022881 | * |
|  | FOS | 1.2393 | ( 1.13, 1.35 ) | 0.003102 | ** |
|  | JUN | 1.8397 | ( 1.69, 1.99 ) | 0.000002 | *** |
| **Target genes for SREBP1** | PCK2 | 0.812 | ( 0.75, 0.88 ) | 0.001783 | ** |
|  | G6PC | 0.9507 | ( 0.88, 1.02 ) | 0.231797 |  |
|  | GCK | 0.9507 | ( 0.88, 1.02 ) | 0.231797 |  |
|  | FBP1 | 0.9774 | ( 0.41, 1.54 ) | 0.873035 |  |
|  | ACACA | 0.991 | ( 0.78, 1.21 ) | 0.9841 |  |
|  | PKM2 | 1.0014 | ( 0.91, 1.09 ) | 0.977038 |  |
| **Carbohydrate metabolism** | SLC2A4 | 0.6641 | ( 0.52, 0.80 ) | 0.007827 | ** |
|  | PCK2 | 0.812 | ( 0.75, 0.88 ) | 0.001783 | ** |
|  | SORBS1 | 0.8612 | ( 0.76, 0.96 ) | 0.04568 | * |
|  | GPD1 | 0.8994 | ( 0.77, 1.03 ) | 0.200002 |  |
|  | SLC2A1 | 0.9119 | ( 0.88, 0.95 ) | 0.00296 | ** |
|  | INSR | 0.9408 | ( 0.92, 0.96 ) | 0.000896 | *** |
|  | G6PC | 0.9507 | ( 0.88, 1.02 ) | 0.231797 |  |
|  | GCK | 0.9507 | ( 0.88, 1.02 ) | 0.231797 |  |
|  | INS | 0.9507 | ( 0.88, 1.02 ) | 0.231797 |  |
|  | FBP1 | 0.9774 | ( 0.41, 1.54 ) | 0.873035 |  |
|  | PPP1CA | 0.9962 | ( 0.85, 1.14 ) | 0.948876 |  |
|  | PKM2 | 1.0014 | ( 0.91, 1.09 ) | 0.977038 |  |
|  | HK2 | 1.0101 | ( 0.92, 1.10 ) | 0.860437 |  |
|  | GSK3B | 1.1227 | ( 1.04, 1.20 ) | 0.01837 | * |
|  | ADRB3 | 1.3009 | ( 0.86, 1.74 ) | 0.20485 |  |
|  | IRS2 | 2.1652 | ( 0.00001, 4.65 ) | 0.324572 |  |
| **Lipid metabolism** | SREBF1 | 0.7708 | ( 0.69, 0.85 ) | 0.00294 | ** |
|  | PRKCG | 0.7749 | ( 0.63, 0.92 ) | 0.037262 | * |
|  | SORBS1 | 0.8612 | ( 0.76, 0.96 ) | 0.04568 | * |
|  | PRKCZ | 0.8672 | ( 0.82, 0.92 ) | 0.002485 | ** |
|  | BRAF | 0.9041 | ( 0.88, 0.93 ) | 0.000521 | *** |
|  | FASN | 0.9056 | ( 0.86, 0.95 ) | 0.012541 | * |
|  | PRKCI | 0.9343 | ( 0.87, 1.00 ) | 0.087155 |  |
|  | ACOX1 | 0.9457 | ( 0.86, 1.03 ) | 0.245107 |  |
|  | RAF1 | 0.949 | ( 0.90, 1.00 ) | 0.117423 |  |
|  | INS | 0.9507 | ( 0.88, 1.02 ) | 0.231797 |  |
|  | ACACA | 0.991 | ( 0.78, 1.21 ) | 0.9841 |  |
|  | SHC1 | 1.0154 | ( 0.95, 1.08 ) | 0.645259 |  |
|  | ARAF | 1.0313 | ( 0.87, 1.19 ) | 0.728604 |  |
|  | PPARG | 1.0677 | ( 1.00, 1.13 ) | 0.077831 |  |
|  | LEP | 1.2874 | ( 1.04, 1.53 ) | 0.040359 | * |
|  | LDLR | 1.404 | ( 1.32, 1.49 ) | 0.000024 | *** |
| **Protein metabolism** | AKT1 | 0.7167 | ( 0.56, 0.88 ) | 0.018722 | * |
|  | PRKCG | 0.7749 | ( 0.63, 0.92 ) | 0.037262 | * |
|  | DOK1 | 0.7994 | ( 0.66, 0.94 ) | 0.038178 | * |
|  | MAP2K1 | 0.8348 | ( 0.79, 0.88 ) | 0.000422 | *** |
|  | SORBS1 | 0.8612 | ( 0.76, 0.96 ) | 0.04568 | * |
|  | PRKCZ | 0.8672 | ( 0.82, 0.92 ) | 0.002485 | ** |
|  | BRAF | 0.9041 | ( 0.88, 0.93 ) | 0.000521 | *** |
|  | ANG | 0.9056 | ( 0.62, 1.19 ) | 0.615926 |  |
|  | DUSP14 | 0.9072 | ( 0.86, 0.95 ) | 0.008593 | ** |
|  | GAB1 | 0.9167 | ( 0.86, 0.98 ) | 0.042123 | * |
|  | MAPK1 | 0.9231 | ( 0.88, 0.97 ) | 0.022478 | * |
|  | AKT3 | 0.9263 | ( 0.83, 1.02 ) | 0.207197 |  |
|  | PRKCI | 0.9343 | ( 0.87, 1.00 ) | 0.087155 |  |
|  | EIF4EBP1 | 0.9343 | ( 0.84, 1.03 ) | 0.231111 |  |
|  | PRKCI | 0.9343 | ( 0.87, 1.00 ) | 0.087155 |  |
|  | INSR | 0.9408 | ( 0.92, 0.96 ) | 0.000896 | *** |
|  | RAF1 | 0.949 | ( 0.90, 1.00 ) | 0.117423 |  |
|  | DOK2 | 0.9507 | ( 0.88, 1.02 ) | 0.231797 |  |
|  | VEGFA | 0.9573 | ( 0.92, 0.99 ) | 0.061682 |  |
|  | EIF2B1 | 0.9589 | ( 0.90, 1.02 ) | 0.234024 |  |
|  | PDPK1 | 0.9673 | ( 0.91, 1.03 ) | 0.344826 |  |
|  | RPS6KA1 | 0.9673 | ( 0.92, 1.02 ) | 0.24085 |  |
|  | INSL3 | 0.9774 | ( 0.45, 1.51 ) | 0.7962 |  |
|  | AEBP1 | 0.9842 | ( 0.84, 1.13 ) | 0.875177 |  |
|  | PTPRF | 0.9859 | ( 0.93, 1.05 ) | 0.648232 |  |
|  | IGF1R | 0.9927 | ( 0.94, 1.05 ) | 0.794456 |  |
|  | PPP1CA | 0.9962 | ( 0.85, 1.14 ) | 0.948876 |  |
|  | PPP1CA | 0.9962 | ( 0.85, 1.14 ) | 0.948876 |  |
|  | CBL | 0.9962 | ( 0.93, 1.06 ) | 0.89076 |  |
|  | GSK3A | 1.0084 | ( 0.95, 1.07 ) | 0.79327 |  |
|  | AKT2 | 1.0207 | ( 0.97, 1.08 ) | 0.477315 |  |
|  | ARAF | 1.0313 | ( 0.87, 1.19 ) | 0.728604 |  |
|  | IRS1 | 1.0882 | ( 0.94, 1.23 ) | 0.266859 |  |
|  | RRAS | 1.1053 | ( 1.05, 1.16 ) | 0.005664 | ** |
|  | NOS2 | 1.113 | ( 0.96, 1.27 ) | 0.177162 |  |
|  | GSK3B | 1.1227 | ( 1.04, 1.20 ) | 0.01837 | * |
|  | FRS2 | 1.1724 | ( 1.09, 1.26 ) | 0.004405 | ** |
|  | PTPN1 | 1.3328 | ( 1.23, 1.44 ) | 0.000476 | *** |
|  | LDLR | 1.404 | ( 1.32, 1.49 ) | 0.000024 | *** |
| **Cell growth and differentiation** | GRB2 | 0.8809 | ( 0.79, 0.97 ) | 0.040764 | * |
|  | GAB1 | 0.9167 | ( 0.86, 0.98 ) | 0.042123 | * |
|  | MAPK1 | 0.9231 | ( 0.88, 0.97 ) | 0.022478 | * |
|  | INSR | 0.9408 | ( 0.92, 0.96 ) | 0.000896 | *** |
|  | MTOR | 0.9474 | ( 0.89, 1.01 ) | 0.1352 |  |
|  | RAF1 | 0.949 | ( 0.90, 1.00 ) | 0.117423 |  |
|  | IGF2 | 0.9507 | ( 0.88, 1.02 ) | 0.231797 |  |
|  | PRL | 0.9507 | ( 0.88, 1.02 ) | 0.231797 |  |
|  | IGFBP1 | 0.9507 | ( 0.88, 1.02 ) | 0.231797 |  |
|  | VEGFA | 0.9573 | ( 0.92, 0.99 ) | 0.061682 |  |
|  | HRAS | 0.969 | ( 0.81, 1.13 ) | 0.724884 |  |
|  | NPY | 0.9825 | ( 0.91, 1.05 ) | 0.631675 |  |
|  | IGF1R | 0.9927 | ( 0.94, 1.05 ) | 0.794456 |  |
|  | NCK1 | 1.0084 | ( 0.93, 1.09 ) | 0.863446 |  |
|  | HK2 | 1.0101 | ( 0.92, 1.10 ) | 0.860437 |  |
|  | SHC1 | 1.0154 | ( 0.95, 1.08 ) | 0.645259 |  |
|  | PPARG | 1.0677 | ( 1.00, 1.13 ) | 0.077831 |  |
|  | NOS2 | 1.113 | ( 0.96, 1.27 ) | 0.177162 |  |
|  | IRS2 | 2.1652 | ( 0.00001, 4.65 ) | 0.324572 |  |

---------------------------------------------------------------------------------------------------------------------------
